# Identification of the Fungal Pathogens of Postharvest Disease on Peach Fruits and the Control Mechanisms of *Bacillus subtilis* JK-14

**DOI:** 10.3390/toxins11060322

**Published:** 2019-06-05

**Authors:** Shuwu Zhang, Qi Zheng, Bingliang Xu, Jia Liu

**Affiliations:** College of Plant protection, Gansu Agricultural University/Biocontrol Engineering Laboratory of Crop Diseases and Pests of Gansu Province, Lanzhou 730070, China; zhangshuw@gsau.edu.cn (S.Z.); zhengshz@126.com (Q.Z.); jiajia7635724@163.com (J.L.)

**Keywords:** *Bacillus* spp., peach fruits, postharvest diseases, antagonistic activity, antioxidative defense system

## Abstract

Postharvest fungal disease is one of the significant factors that limits the storage period and marketing life of peaches, and even result in serious economic losses worldwide. Biological control using microbial antagonists has been explored as an alternative approach for the management of postharvest disease of fruits. However, there is little information available regarding to the identification the fungal pathogen species that cause the postharvest peach diseases and the potential and mechanisms of using the *Bacillus* *subtilis* JK-14 to control postharvest peach diseases. In the present study, a total of six fungal isolates were isolated from peach fruits, and the isolates of *Alternaria tenuis* and *Botrytis cinerea* exhibited the highest pathogenicity and virulence on the host of mature peaches. In the culture plates, the strain of *B. subtilis* JK-14 showed the significant antagonistic activity against the growth of *A. tenuis* and *B. cinerea* with the inhibitory rates of 81.32% and 83.45% at 5 days after incubation, respectively. Peach fruits treated with different formulations of *B. subtilis* JK-14 significantly reduced the mean disease incidences and lesion diameters of *A. tenuis* and *B. cinerea*. The greatest mean percent reduction of the disease incidences (81.99% and 71.34%) and lesion diameters (82.80% and 73.57%) of *A. tenuis* and *B. cinerea* were obtained at the concentration of 1 × 10^7^ CFU mL^−1^ (colony forming unit, CFU). Treatment with the strain of *B. subtilis* JK-14 effectively enhanced the activity of the antioxidant enzymes-superoxide dismutase (SOD), peroxidase (POD) and catalase (CAT) in *A. tenuis* and *B. cinerea* inoculated peach fruits. As such, the average activities of SOD, POD and CAT were increased by 36.56%, 17.63% and 20.35%, respectively, compared to the sterile water treatment. Our results indicate that the isolates of *A. tenuis* and *B. cinerea* are the main pathogens that cause the postharvest peach diseases, and the strain of *B. subtilis* JK-14 can be considered as an environmentally-safe biological control agent for the management of postharvest fruits diseases. We propose the possible mechanisms of the strain of *B. subtilis* JK-14 in controlling of postharvest peach diseases.

## 1. Introduction

Postharvest losses refer to the losses that occur along the food supply chain due to pathogens infection, handling, storage, transportation and processing, thereby resulting in the reduction in quality, quantity and market value of agricultural commodities [1,2]. Food and Agriculture Organization reported that global average loss due to the food postharvest losses in North America, Europe and Oceania was about 29%, compared to an average of about 38% in industrialized Asia, Africa, Latin America and South East Asia [3]. Among all the factors for reducing the losses on food supply, postharvest diseases of fruits present a major factor that causes the postharvest losses and limits the duration of storage [4,5]. In addition, postharvest diseases are often the major concern in influencing consumer prices, requirements and mode of transportation [6]. China is the largest producer of peaches with a production of 13.5 million metric tons (MMT), and exporter to North Korea, Russia, Singapore, USA, Philippines and Malaysia, with a very different climate (from tropical, continental or oceanic climatic climates), but the postharvest diseases of peach fruits have been considered one of the most severe factors that results in the loss of production [7]. Additionally, the diseases caused by fungal pathogens in harvested fresh fruits are considered as one of the most serious losses of production at the postharvest and consumption levels [8,9,10]. Some research showed that the main worldwide postharvest diseases caused by fungi in peach fruits are brown rot caused by *Monilinia fructicola* or *M. laxa*, *Rhizopus*; rot caused by *R. stolonifer*; grey mold caused by *Botrytis cinerea* [11], and other economically important fungal diseases such as those of stone fruits caused by *Penicillium* spp., *Cladosporium* spp., *Alternaria* spp. and *Aspergillus* spp. [12,13,14]. However, little is known about the species of main fungal pathogens that cause the postharvest disease of peaches in China.

A number of strategies have been adapted to manage of postharvest diseases worldwide [15,16]. Chemical control (synthetic fungicides) is known to be highly effective and widely applied method in orchard after harvesting [17,18]. However, some fungicides have toxicological risks, such as dangerous to human health and causing environmental pollution, even in some cases their use is prohibited by law in postharvest phase [19,20,21]. Particularly, the increased level of fungicide use in fruit orchards has led to the growing public concern over the health and environmental hazards associated with fungicides [22]. Therefore, development of alternative safe and natural methods in controlling postharvest diseases have become urgent in recent years worldwide [23,24]. In particular, there has been extensive research to reduce synthetic fungicide usage based on microbial antagonists to biologically control postharvest pathogens in the past years with higher control efficiency [10,15,23,25]. In recent years, the bacterium *Bacillus* spp. has been widely studied as a potential biological agent against various plant diseases, increases plant systemic resistance and improves rhizosphere microbial community structure [26,27,28,29]. It is common in nature and nontoxic and harmless to humans and other animals, and nonpathogenic to plants [30]. However, there is little information on the bio-control activity of the bacterial antagonist *B. subtilis* JK-14 and its mechanisms involved in the postharvest disease management of peaches.

Therefore, the objectives of the present study were to (i) isolate and identify the main species of fungal pathogens causing postharvest disease on peaches, (ii) explore the antifungal potential and controlling efficiency of *B. subtilis* JK-14 against the main postharvest fungal infection, and (iii) determine the possible mechanisms involved in the strain of *B. subtilis* JK-14 in controlling postharvest fruit diseases on peaches.

## 2. Results

### 2.1. Isolation and Identification of Postharvest Fungal Pathogens

**Figure 1:**
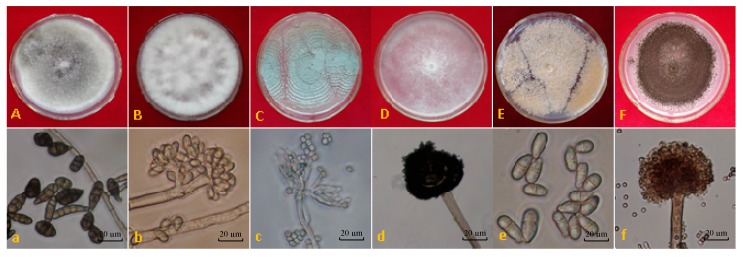
Characteristics of colony (A–F) and conidia (a–f) of the pathogenic isolates of peaches grew on potato dextrose agar (PDA) for 5 days. Where (**A**) and (**a**) the colony and conidia of *Alternaria tenuis*; (**B**) and (**b**) *Botrytis cinerea*; (**C**) and (**c**) *Penicillium digitatum*; (**D**) and (**d**) *Rhizopus nigricans*; (**E**) and (**e**) *Trichothecium roseum*; (**F**) and (**f**) *Aspergillus niger*.

In the present study, a total of six fungal isolates were isolated from the mature peach (*Prunus persica* L.) fruits during the storage period. They were identified as *Alternaria tenuis* (Figure 1A,a), *Botrytis cinerea* (Figure 1B,b), *Penicillium digitatum* (Figure 1C,c), *Rhizopus nigricans* (Figure 1D,d), *Trichothecium roseum* (Figure 1E,e), and *Aspergillus niger* (Figure 1F,f), respectively, according to the characteristics of colony (Figure 1A–F) and conidia (Figure 1a–f) under the microscope observation. The isolates of *A. tenuis*, *B. cinerea*, *P. digitatum*, *T. roseum*, *A. niger*, and *R. nigricans* were all grown better on potato dextrose agar (PDA) medium (pH = 6.0) at 25 °C as the ecophysiological conditions. 

### 2.2. Determination of the Pathogenicity of the Isolates

The difference in disease incidences of infection caused by the six fungal isolates on the mature fruits was highly significant between intact and wounded fruits. The non-inoculated control fruits, including intact and wounded did not develop decay symptoms. In contrast, all the wounded fruits developed rot and decay, regardless of the isolates used. Particularly, the isolates of *A. tenuis*, *B. cinerea* and *R. nigricans* presented the highest disease incidences after inoculation onto the wounded fruits, and the disease incidences were all 100%. In addition, the highest disease incidences were observed after inoculation with the isolates of *A. tenuis* and *B. cinerea* on the intact fruits, and the disease incidences were 100% and 92.33%, respectively. Whereas the isolate of *T. roseum* was unable to infect the intact fruits under the same experimental conditions (Table 1). 

### 2.3. Inhibitory Effect of Bacillus subtilis JK-14 against Alternaria tenuis and Botrytis cinerea

Our results showed that the strain of *B. subtilis* JK-14 presented the significant antagonistic activity on the pathogens of *A. tenuis* and *B. cinerea* compared to the control. In the culture plates (PDA), the colony growth of *A. tenuis* and *B. cinerea* were significantly inhibited at 5 days after inoculated with the antagonistic strain of *B. subtilis* JK-14. The inhibitory rates of *A. tenuis* and *B. cinerea* were 81.32% and 83.45% at 5 days after inoculation with the strain of *B. subtilis* JK-14, respectively (Table 2).

**Figure 2:**
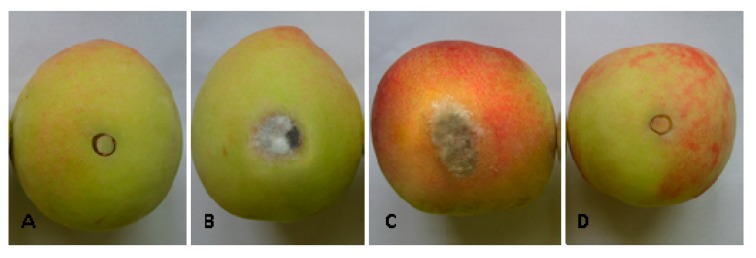
Inhibitory activities of *Bacillus subtilis* JK-14 in controlling *Alternaria tenuis* and *Botrytis cinerea* on fresh peaches. (**A**) *Bacillus subtilis* JK-14 and *Alternaria tenuis* inoculation; (**B**) *Alternaria tenuis* inoculation; (**C**) *Botrytis cinerea* inoculation; (**D**) *Bacillus subtilis* JK-14 and *Botrytis cinerea* inoculation.

To further confirm the antagonistic activity of *B. subtilis* JK-14 in controlling the *A. tenuis* and *B. cinerea* decay on fresh peaches fruits. We used the bacterial cell suspension (BCS) of *B. subtilis* JK-14 treatment and found that it was effective in inhibiting the fresh fruits decay caused by the pathogens of *A. tenuis* and *B. cinerea*, compared to the control. The disease incidences and lesion diameters on the peach fruits treated with the BCS of *B. subtilis* JK-14 at the tested concentration of 1 × 10^8^ CFU mL^−1^ were significantly reduced compared to those on the control fruits. The disease incidences and lesion diameters were 14.8% and 3.0 mm for *A. tenuis*, and 14.1% and 3.2 mm for *B. cinerea* after 5-day incubation, whereas the average disease incidences and lesion diameters of *A. tenuis* and *B. cinerea* decay in the control fruits were 93.7% and 12.6 mm, respectively (Table 3). In addition, there was no significant symptoms and mycelium around the inoculation site of fresh fruits after inoculated with the pathogen of *A. tenuis* and the antagonist of *B. subtilis* JK-14 (Figure 2A), and the pathogen of *B. cinerea* and *B. subtilis* JK-14 together (Figure 2D) in treatment group, whereas the fruits in the control group were decayed significantly and a large number of hyphae grown around the wound sites, regardless of the pathogens of *A. tenuis* (Figure 2B) and *B. cinerea* (Figure 2C) that was inoculated alone in this experiment without *B. subtilis* JK-14.

### 2.4. Effect of Bacillus subtilis JK-14 in Controlling Alternaria tenuis and Botrytis cinerea Decay on Peaches

The disease incidences and lesion diameters of postharvest decay of peaches treated with the different formulations of *B. subtilis* JK-14 at all tested concentrations (1 × 10^5^–1 × 10^9^ CFU mL^−1^) were significantly reduced compared to those of the control fruits for the decay caused by the pathogens of *A. tenuis* (Table 4) and *B. cinerea* (Table 5). Among all the different concentrations of the formulations, the disease incidences and lesion diameters of postharvest decay of peaches were significantly reduced by the application of fermentation liquid bacterial cells (FLBC) and BCS of *B. subtilis* JK-14 at 1 × 10^7^ CFU mL^−1^. The disease incidences and lesion diameters were 18.52% and 3.79 mm for *A. tenuis* and 17.78% and 3.74 mm for *B. cinerea* at 5 days after inoculated with the FLBC formulations of *B. subtilis* JK-14 at 1 × 10^7^ CFU mL^−1^, 14.82% and 3.06 mm for *A. tenuis* and 14.07% and 3.19 mm for *B. cinerea* after inoculated with the BCS. In contrast, the disease incidences and lesion diameters of *A. tenuis* decay in the control fruits were 92.59% and 11.95 mm, respectively (Table 4), and 92.59% and 13.11 mm, respectively, of *B. cinerea* decay in the control fruits (Table 5).

In addition, the controlling effect of different formulations of the strain of *B. subtilis* JK-14 was different between the formulations of FLBC and BCS at all the tested concentrations. The average disease incidences and lesion diameters of *A. tenuis* and *B. cinerea* decay of the fruits treated with the BCS formulations were lower than the formulation of FLBC. Therefore, the BCS formulations of the strain of *B. subtilis* JK-14 exhibited the highest controlling effect on the *A. tenuis* and *B. cinerea* decay compared to the control (Table 4 and Table 5).

### 2.5. Effect of Bacillus subtilis JK-14 on the Symptoms of Fruits Decay after Inoculation with the Pathogens on Peaches

**Figure 3:**
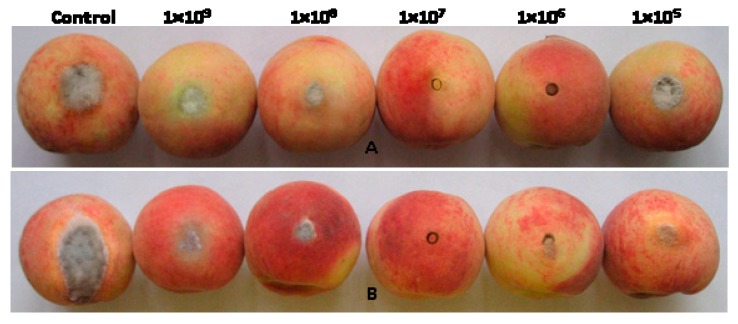
Effect of *Bacillus subtilis* JK-14 on the symptoms of peach fruits decay at 5 days after inoculation with the pathogens of (**A**) *Alternaria tenuis* and (**B**) *Botrytis cinerea.*

Overall, the different concentrations of BCS formulation of *B. subtilis* JK-14 (1 × 10^5^, 1 × 10^6^, 1 × 10^7^, 1 × 10^8^, and 1 × 10^9^ CFU mL^−1^) had the different inhibitory and controlling effects on the *A. tenuis* (Figure 3A) and *B. cinerea* (Figure 3B) decay of the fruits. The BCS formulation of *B. subtilis* JK-14 at the concentration of 1 × 10^7^ CFU mL^−1^ had a stronger and more significant inhibitory and controlling effect (Figure 3). At the concentrations of 1 × 10^5^, 1 × 10^8^ and 1 × 10^9^ CFU mL^−1^, the fruits were decayed significantly and a large number of hyphae grown on the surface. At the concentration of 1 × 10^6^ CFU mL^−1^, the fruits became decay and a fewer number of hyphae grew on the surface. However, there was no significant symptoms and mycelium around the inoculation site at the concentration of 1 × 10^7^ CFU mL^−1^. In contrast, the fruits were exhibited significant decay and a large number of hyphae grew around the wound site in the untreated control fruits.

### 2.6. Effect of Bacillus subtilis JK-14 on the Activities of Defense-Related Enzymes of Peaches

**Figure 4:**
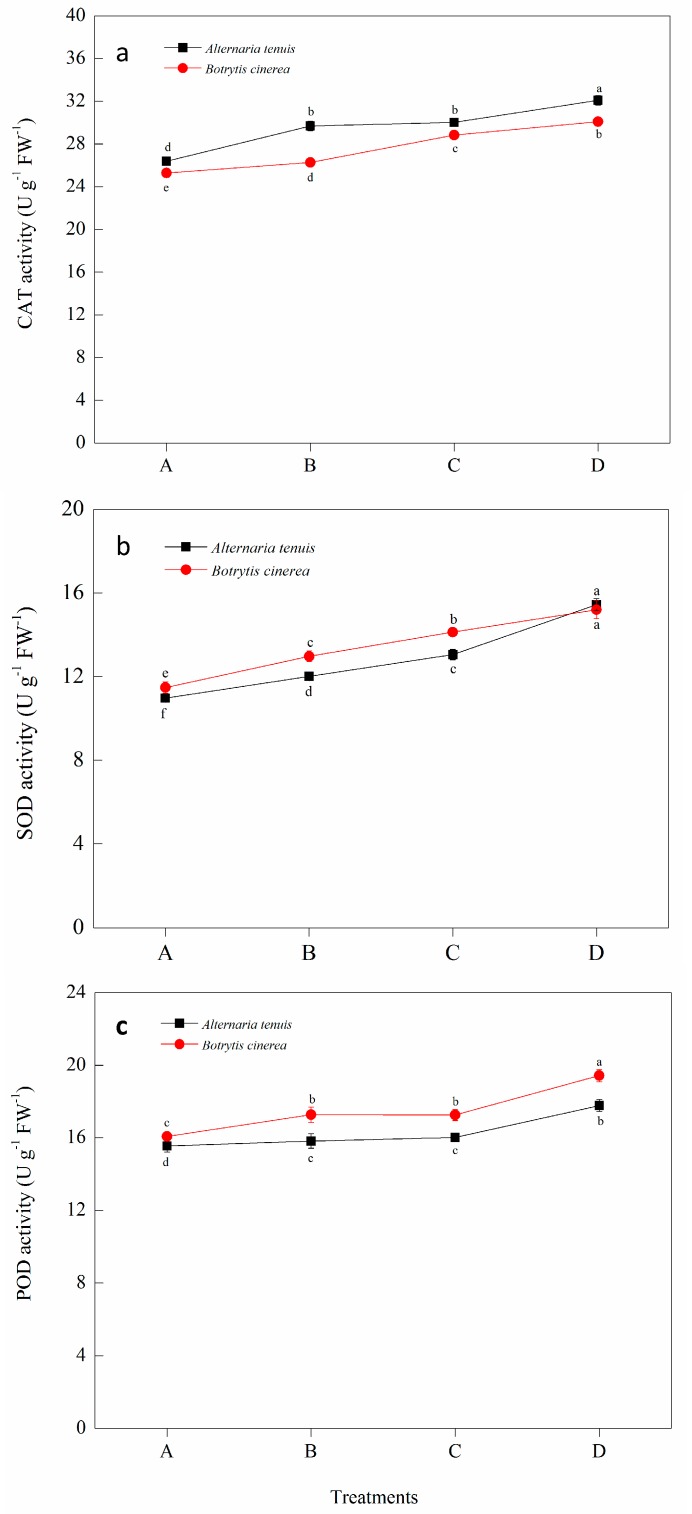
Effect of *Bacillus subtilis* JK-14 on the activity of (**a**) CAT, (**b**) SOD and (**c**) POD of peach fruits after inoculation with the pathogens of *Alternaria tenuis* and *Botrytis cinerea.* A—sterile water, B—pathogens inoculation, C—the strain of *Bacillus subtilis* JK-14 inoculation, D—*Bacillus subtilis* JK-14 and pathogens inoculation. Each value is the mean of two experiments. Line bars represent the standard errors of the means. Data in columns with the different letters are significantly different according to Duncan’s multiple range test at *p < 0.05* (*n* = 18).

The effects of *B. subtilis* JK-14 on the activities of defense-related enzymes of peaches fruits were determined after inoculation with the pathogens of *A. tenuis* and *B. cinerea* onto the fruits. Our results found that the pathogens (*A. tenuis* and *B. cinerea*) or the *B. subtilis* JK-14 alone induced and increased the defense-related enzymes activities in peach fruits, including the activities of CAT, SOD and POD (Figure 4) in comparison to the sterile water treatments. Moreover, the activities of CAT, SOD and POD were significantly increased after being treated with the strain of *B. subtilis* JK-14, and the pathogens of *A. tenuis* or *B. cinerea* onto fruits, compared to the sterile water, *B. subtilis* JK-14, and the pathogens-inoculated fruits alone. Compared to the sterile water (Treatment A), the activity of CAT (Figure 4a), SOD (Figure 4b) and POD (Figure 4c) were significantly increased by 21.67%, 40.69% and 14.32%, respectively, on the 4th day after inoculation with the strain of *B. subtilis* JK-14 and the pathogen of *A. tenuis* (Treatment D) and 19.04%, 32.43% and 20.93%, respectively, after the inoculation with *B. subtilis* JK-14 and the pathogen of *B. cinerea* (Treatment D). In addition, the *B. subtilis* JK-14 and *A. tenuis* (Treatment D) treatment increased the activity of CAT (Figure 4a), SOD (Figure 4b) and POD (Figure 4c) by 8.09%, 28.55% and 12.39% on the 4th day, respectively, and 14.58%, 17.19% and 12.53%, respectively, after treatment with *B. subtilis* JK-14 and *B. cinerea* (Treatment D) in comparison to the pathogens inoculation (Treatment B). Moreover, the activity of POD in seedlings treated with *B. subtilis* JK-14 alone (Treatment C) did not differ from fruits treated with the pathogens treatments (Treatment B) (Figure 4c), whereas the activities of SOD differed significantly between the two treatments (Treatment B and C) after inoculation with *A. tenuis* or *B. cinerea* (Figure 4b).

## 3. Discussion and Conclusions

Peach is one of the most ancient and world-popular fruits due to its high marketing value with favorable taste and abundant phytonutrients [31]. However, postharvest fungal diseases limit the storage period and marketing life of peaches, and result in serious economic losses worldwide. Recently, application of bio-control agents for the management of postharvest fruit decay has been explored as an alternative method instead of synthetic fungicides worldwide [15]. *Bacillus* spp. has been considered as the bio-control agent in controlling number of plant diseases with a high efficacy [32,33]. However, there is little information available regarding the identification the fungal pathogen species that cause the peach postharvest diseases, and explore the potential and mechanisms of *Bacillus subtilis* JK-14 in controlling postharvest peach diseases. Our present study showed that a total of six fungal isolates were isolated from the mature peaches, and in particular the species of *Alternaria tenuis* and *Botrytis cinerea* have been identified as the main pathogens for causing the host of mature peach decay. Interestingly, the strain of *B. subtilis* JK-14 has been found and exhibited a potent activity in inhibiting the growth of *A. tenuis* and *B. cinerea*, and controlling peaches fruits fungal disease in the present study. The possible mechanisms for the strain of *B. subtilis* JK-14 in inhibiting and controlling postharvest peaches fungal disease were due to the direct effect by inhibiting the pathogens infection, and the indirect effect by activating the host defense response to pathogens infection. To the best of our knowledge, the present study is the first to discover the role of the antagonistic *B. subtilis* JK-14 in controlling peach fungal disease that are caused by the pathogens of *A. tenuis* and *B. cinerea*. In view of the high control efficacy in comparison to the control, the strain of *B. subtilis* JK-14 can be considered as an environmentally-safe biological control agent instead of chemical fungicides for the management of postharvest disease. 

Some previous studies found and identified numerous postharvest pathogens which can cause the decay of stone fruits and belong to the genera of *Monilinia*, *Rhizopus*, *Penicillium*, *Alternaria*, *Botrytis*, *Cladosporium*, *Colletotrichum* and *Stigmina* [34], *Trichothecium* [35] and *Aspergillus* [36]. Interestingly, six fungal isolates were isolated from the mature peach fruits in the present study, including *A. tenuis*, *B. cinerea*, *P. digitatum*, *T. roseum*, *R. nigricans* and *A. niger*. Our results confirm for the first time that these species are pathogenic to peach fruit and cause decay on wounded peach fruits. However, we have discovered that the isolate of *T. roseum* was not pathogenic to the intact peach fruits. The reason may due to the lack of wounds that prevent the *T. roseum* invasion. A similar study demonstrated that the wounds can provide the pathways for the pathogens invasion [25]. In addition, some previous studies revealed that the gray mold decay, blue mold decay and *Rhizopus* decay caused by the fungi of *B. cinerea*, *P. expansum* and *R. stolonifer* were the most economically significant and destructive postharvest diseases of peaches [5,8,37,38]. However, our results found that the isolates of *A. tenuis* and *B. cinerea* presented the highest pathogenicity and virulence on the host of mature peaches, and also considered as the main pathogens that cause the postharvest disease of peach fruits. The average disease incidences of *A. tenuis* and *B. cinerea* were 100% and 96.17% after inoculation onto the wounded and intact fruits, respectively. The difference from the previous studies may due to the relationship between the pathogenicity of microbial isolates and the ripening index of peach fruits at harvest [39,40].

In view of the need for reducing environmental pollution due to fungicide over-use in controlling plant diseases in previous years, recently, biological control has emerged as an effective strategy to combat major postharvest decay of fruits [25,41]. It is well-known that *B. subtilis* is an effective antagonistic bacterium and has been applied in controlling plant fungal diseases such as root diseases [42], foliar diseases [43] and postharvest diseases [15]. A significant advancement from the present study is the finding that *B. subtilis* JK-14 provided a significant inhibitory effect on the peach fruits pathogens of *A. tenuis* and *B. cinerea*, and also different formulations of *B. subtilis* JK-14 exhibited significant controlling effect on the peach fruits decay after inoculation with pathogens of *A. tenuis* and *B. cinerea.* Our findings suggest that the strain of *B. subtilis* JK-14 can be considered as a bio-control agent in the effort of developing alternative approaches to control postharvest diseases of fruits.

A previous study showed that *Bacillus* sp. C06 suppressed the disease incidences of the postharvest disease brown rot by 92% and decreased the lesion diameters by 88% compared to the pathogen-only, and *Bacillus* sp.T03-c reduced disease incidences and lesion diameters by 40% and 62%, respectively [44]. Similarly, Xu et al. reported that the treatment with *Pichia caribbica* significantly reduced the disease incidences and lesion diameters of *Rhizopus* decay of peaches compared with the control fruits in a dose dependent manner [7]. However, our results revealed that the greatest mean percent reduction of disease incidences and lesion diameters of peach postharvest fungal disease by 82.40% and 72.46% after the application of *B. subtilis* JK-14 at 1 × 10^7^ CFU mL^−1^ among all the different concentrations from 1 × 10^5^ to 1 × 10^9^ CFU mL^−1^. Such differences may be related to the effect of the species of pathogens and different conditions of ripening index of peach fruits (pH value) at harvest on the inhibitory effect of *B. subtilis* JK-14 [39]. 

To further understand the mechanisms of *B. subtilis* JK-14 in controlling postharvest diseases of peaches, we explored the effects of *B. subtilis* JK-14 on the activities of defense-related enzymes after inoculation with the pathogens in the present study, and found that the treatment of peach fruits with *B. subtilis* JK-14 effectively enhanced the activities of superoxide dismutase (SOD), peroxidase (POD) and catalase (CAT) after inoculation with the pathogen of *A. tenuis* or *B. cinerea*. Our results indicate that the enhanced activities of defense-related enzymes may play a significant role in the resistance of peaches to the pathogens infection and the induced activity of defense-related enzymes to be part of the mechanism of *B. subtilis* JK-14 in controlling postharvest diseases of peach fruits. Some previous studies revealed that one of the important mechanisms for the genus *Bacillus* in controlling plant diseases is by increasing and activating the plant systemic resistance [45,46,47]. In addition, the enhanced activities of antioxidant enzymes (SOD, POD, CAT and ascorbate peroxidase, APX) and their coordinated action have been reported to be a part of the mechanism implicated in the alleviation of lipid peroxidation and delay of senescence in peach fruits [48]. Similarly, Xu et al. [7] have demonstrated that peach fruits inoculated with *P. caribbica* exhibited higher level of POD, CAT and phenylalanine aminolase (PAL) activities than the untreated fruits during the storage period. 

In summary, a total of six isolates were isolated from the peach fruits, and the isolates of *A. tenuis* and *B. cinerea* were considered as the main pathogens with the highest pathogenicity and virulence on the host of mature peaches. The strain of *B. subtilis* JK-14 exhibits a high efficacy in controlling postharvest decay of peaches, and may be considered as an environmentally-safe biological control agent for the management of postharvest decay diseases. The possible mechanisms of *B. subtilis* JK-14 for the management of peach postharvest disease were due to (i) the direct effect by inhibiting the postharvest fungal pathogens growth and infection, and (ii) the indirect effect by activating the defense-related enzymes to enhance the resistance of peaches response to the postharvest fungal pathogens infection during the storage period. 

## 4. Materials and Methods

Experiments were carried out at the Gansu Provincial Biocontrol Engineering Laboratory of Crop Diseases and Pests. The peach (*Prunus persica* L., cultivar Baifeng) fruits were collected from the stone fruit orchards in Gansu, China. Gansu is located in the northwest of China, at the longitude of 103.8264470 E and latitude of 36.0595610 N, with a dry and strong continental temperate monsoon climate. The average temperature, precipitation and relative humidity of the air were about 8 °C, 300 mm and 30% in 2011–2012.

### 4.1. Fungal Pathogens Isolation and Identification

During 2011–2012, the mature peach (cultivar Baifeng) fruits were collected from the stone fruit orchards in Gansu, China. The ripening index of peach fruits at harvest: pH 3.75–3.98, organic acid 2.28–2.64 mg g^−1^, ethylene production 16.23–21.46 µL kg^−1^ h^−1^, soluble solids content 12.24–13.16%, total sugar 90.85–110.90 mg g^−1^, pectic substances 9.2–13.8 mg g^−1^. Thereafter, fruits were moist-incubated by placing in plastic containers with lids, lined with moist paper towels to maintain high relative humidity, and incubated at room temperature (20 °C) for 1–2 weeks to promote the pathogens growth and development. Small fruits sections (2 cm) were surface sterilized with 2% sodium hypochlorite (NaClO) for 3 min, and followed by 3 min rinses in sterile water. Fruits were then cut lengthwise along the lesion (1 cm) and placed individually onto PDA for 5 days at 25 °C. The spores and mycelium were transferred with a sterile needle from the colony to fresh Petri dishes containing PDA medium at Day 5. These cultures were grown for 5 days in an incubator at 25 °C, and then identified according to the colony and spores characteristics. Finally, all isolates were maintained and stored in 20% glycerol at −80 °C until use. 

### 4.2. Spore Suspensions of Fungal Pathogen Preparation

The identified pathogens of peaches were cultured on PDA medium for 5 days, and then suspended in 5 mL of sterile water containing 0.05% (*v*/*v*) Tween-80. Thereafter, the spore suspensions were filtered through 0.22 mm Millipore membranes to remove any adhering mycelia. The concentration of the spore suspension was determined using a hemacytometer, and then, the final concentration was adjusted to 1 × 10^6^ CFU mL^−1^ [49].

### 4.3. Fruit Preparation

For inoculum production, the experiments were conducted with the peach (*Prunus persica* L.) fruit cultivars Baifeng. The fresh fruits (pH = 3.53–3.64) were collected one week before commercial harvest during the 2012 production season, and the mature fruits (pH = 3.75–3.98) were collected and harvested at the mature stage, and sorted based on the size and the absence of physical injuries or disease infection. Before treatments, fruits were disinfected on the surface with 2% (*v*/*v*) NaClO for 3 min, and then rinsed with sterile water and air-dried for approximately 30 min at room temperature (20 °C) prior to use [50] and inoculation.

### 4.4. Pathogenicity of the Isolates on Peach Fruits

All the isolates in the present study were tested for pathogenicity on the mature peach fruits. Two groups of treatments were designed in this experiment, (i) one group of the sterile fruits were wounded once to a depth of 3 mm with a sterilized needle in the equatorial zone (wounded fruits) and (ii) another group of the sterile fruits were non-wounded with a sterilized needle (intact fruits). A 5-mm-diameter plug from a 5-day-old mycelial culture of isolates was inoculated onto intact and wounded peach fruits. Additionally, a 5-mm-diameter PDA plug was used as the untreated control treatment. Thereafter, all the treatments fruits were moist-incubated by placing in plastic containers with lids, lined with moist paper towels to maintain high relative humidity and incubated at room temperature (20 °C). Pathogenicity was determined as the ability to cause the typical decay symptom, and the number of fruit infected. The parameter of disease incidences was measured at 5 days after inoculation. Each experiment had three replications and each replication had three fruits, and all the experiments were repeated twice. The highest pathogenic pathogens were used to determine the antagonistic activity of *Bacillus subtilis* JK-14 in later experiments.

### 4.5. Formulations of Bacillus Subtilis JK-14 Preparation

The strain of *B. subtilis* JK-14 used in the present study was obtained from the College of Plant Protection, Gansu Agricultural University, isolated from the surface of peach fruits from an orchard in Gansu, China, and tested for its antifungal potential against the highest pathogenicity of the isolates on mature peach fruits. The active colony was then prepared by culturing on nutrient agar (NA, pH = 7.0) in Petri dishes for 3 days at 28 °C. A culture of *B. subtilis* JK-14 was obtained by transferring a colony from the activated culture plate into a 150 mL flask containing 30 mL liquid broth (peptone 0.3 g, yeast extract 0.3 g, NaCl 0.05 g) and shaking in an orbital shaker (200 rpm min^−1^) at 28 °C for 48 h. A formulation of fermentation liquid with bacterial cells (FLBC) was made by incubating the bacterial culture under the same conditions, and then dissolved with the sterile water to prepare the final concentrations of FLBC from 1 × 10^5^ to 1 × 10^9^ CFU mL^−1^. A formulation of bacterial cell suspension (BCS) was prepared by centrifuging the fermentation liquid at 12, 000 rpm min^−1^ at 4 °C for 20 min, and filtered by 0.22 µm biofilter to collect the bacterial sediment. Thereafter, the bacterial sediment was washed with an equal volume of saline (0.85% NaCl), and then dissolved with the sterile water to prepare the final concentrations of BCS from 1 × 10^5^ to 1 × 10^9^ CFU mL^−1^. The two formulations were stored at 4 °C for later use.

### 4.6. In Vitro and in Vivo Antagonistic Activity Determination

In vitro experiments, the antagonistic activity of *B. subtilis* JK-14 against the main pathogens, were conducted following dual culture plate technique [51]. The inhibitory effects of *B. subtilis* JK-14 on the isolates with the highest pathogenicity were done by examining the growth rates inhibition using the paper–disc method on PDA [52,53]. Each experiment had six replications and was repeated twice.

For the confirmation of the antagonistic activity of *B. subtilis* JK-14 (BCS formulation) in controlling *A. tenuis* and *B. cinerea* decay in fresh peach wounds, the fruits experiments were conducted to determine the controlling effects in vivo. A uniform wound (3 mm diameter and 3 mm deep) was made at the equator of each peach fruit using sterilized needle. An aliquot (30 µL) of *B. subtilis* JK-14 at 1 × 10^8^ CFU mL^−1^ was pipetted into each wound site, and 30 µL of sterile water in place of the *B. subtilis* JK-14 was used as the control. Two hours later, 15 µL spores suspension of *A. tenuis* and *B. cinerea* (1 × 10^6^ CFU mL^−1^) were inoculated into each wound, respectively. After air drying, the peaches were stored in enclosed plastic containers to maintain a high relative humidity (RH 85%) at 20 °C. Disease incidences and lesion diameters, and the symptoms of the treated peach fruits were measured and observed at 5 days after inoculation. All treatments were carried out with three replicates and three fruits for each treatment, and the experiment was conducted twice.

### 4.7. Efficacy of Bacillus subtilis JK-14 in Controlling of Peach Postharvest Disease

For the fruits inoculation, peach fruit samples were treated as described above to determine the antagonistic activity of *B. subtilis* JK-14 formulations (FLBC and BCS) in inhibiting *A. tenuis* and *B. cinerea* decay in mature peach wounds in vivo. An aliquot (30 µL) of different formulations of *B. subtilis* JK-14 at 1 × 10^5^, 1 × 10^6^, 1 × 10^7^, 1 × 10^8^, and 1 × 10^9^ CFU mL^−1^ was pipetted into each wound site, and 30 µL of sterile water in place of the *B. subtilis* JK-14 formulations was used as the control. Two hours later, 15 µL spores suspension of *A. tenuis* and *B. cinerea* (1 × 10^6^ CFU mL^−1^) were inoculated into each wound, respectively. After air drying, the incubation condition of treated peaches as described above. Disease incidences and lesion diameters, and the symptoms of the treated mature peach fruits were measured and observed at 5 days after inoculation. All treatments were carried out with three replicates and three fruits for each treatment, and the experiment was conducted twice.

### 4.8. Effects of Bacillus subtilis JK-14 on the Activities of Defense-Related Enzymes of Peaches

Peach fruit samples were treated as described above to test the efficacy of *B. subtilis* JK-14 in inhibiting *A. tenuis* and *B. cinerea* decay in mature peach wounds. The wounds were then treated with 30 µL of BCS of *B. subtilis* JK-14 at 1 × 10^7^ CFU mL^−1^, and 30 µL of sterile water in place of the BCS formulation of *B. subtilis* JK-14 was used as the control. Two hours later, 15 µL spores suspension of the highest pathogenicity of the isolates of *A. tenuis* and *B. cinerea* (1 × 10^6^ CFU mL^−1^) were inoculated into each wound. The treatments of sterile water and *B. subtilis* JK-14 alone were considered as the controls. The peach fruits were stored in enclosed plastic containers to maintain a high relative humidity (RH 85%) and incubated at 20 °C after air drying. In order to measure the activities of defense-related enzymes of peaches after the treatment of *B. subtilis* JK-14, the tissue surrounding each wound of fruit was collected at Day 4 after treatment. Three replicates consistent of three fruits were sampled in both inoculated group and control group, and the experiments were conducted twice.

### 4.9. Determination and Analysis of Defense-Related Enzyme Activities of Peaches

The extraction procedures of the enzyme extract from the collected samples were conducted following the method of Xu et al. [7]. The tissue surrounding each wound of fruits (2 g) were collected and homogenized with 4 mL of ice-cold sodium phosphate buffer (50 mM, pH 7.8) containing 1.33 mM EDTA and 1% PVP. Thereafter, the homogenates were then centrifuged at 12,000× *g* for 15 min at 4 °C, and the supernatants were collected and used as enzyme extract to assay the activity of POD, SOD and CAT of peaches after extraction using the spectrophotometer (AOE (UV1900), Shanghai, China).

POD activity was assayed following the method of Meng et al., with some minor modifications [54]. The reaction mixture containing 0.2 mL of the enzyme extract and 2.2 mL of 0.3% guaiacol was incubated for 5 min at 30 °C, and then the reaction was initiated immediately by adding 0.6 mL of 0.3% H_2_O_2_. The activity of POD was determined by measuring absorbance at 470 nm, and expressed as U per g fresh weight (U g^−1^ FW^−1^).

SOD activity was measured following the method of Giannopolitis and Ries, and determined by assaying the ability to inhibit the photochemical reduction of nitroblue tetrazolium chloride (NBT) [55]. The reaction mixture (1.5 mL) contained 50 mM phosphate buffer (pH 7.8), 0.1 µM EDTA, 13 mM methionine, 75 µM NBT, 2 µM riboflavin and 50 µL enzyme extracts. One unit of SOD activity was defined as the amount of enzyme required to cause 50% inhibition of the NBT photo reduction rate, and the results were expressed as U g^−1^ FW^−1^.

CAT activity was measured according to the method described by Wang et al. [56], with some modifications. The reaction mixtures contained 1.4 mL buffered substrate (50 mM sodium phosphate, pH 7.8, and 30 mM H_2_O_2_) and 100 µL of enzyme extracts. The decomposition of H_2_O_2_ was measured by the decline in absorbance at 240 nm. One unit of the CAT activity was defined as the amount of the H_2_O_2_ decomposing, and the activity was expressed as U g^−1^ FW^−1^. 

### 4.10. Statistical Analysis

Data presented in the present paper were pooled across two independent repeated experiments. All statistical analyses were performed with SPSS version 16.0 (SPSS Inc., Chicago, IL, USA, 2007). Data were analyzed by multi-way ANOVA. Duncan’s multiple range test were computed using standard error and T values of adjusted degrees of freedom. Differences at *p* < 0.05 were considered significant.

## Figures and Tables

**Table 1:** The pathogenicity of six fungal isolates after inoculation onto the postharvest peach fruits.

Isolates	Disease Incidences (%)	
	Wound Inoculation	Intact Inoculation
*Alternaria tenuis*	100.00 ± 0.00 a	100.00 ± 0.00 a
*Botrytis cinerea*	100.00 ± 0.00 a	92.33 ± 3.16 c
*Penicillium digitatum*	95.46 ± 4.21 b	56.67 ± 3.02 f
*Trichothecium roseum*	34.54 ± 2.56 g	0.00 ± 0.00 i
*Rhizopus nigricans*	100.00 ± 0.00 a	83.33 ± 2.89 d
*Aspergillus niger*	67.63 ± 2.33 e	17.34 ± 1.54 h
Control	0.00 ± 0.00 i	0.00 ± 0.00 i

Data are means ± standard error of replicates and those in a column followed by different letters are significantly different at *p* < 0.05, based on Duncan’s new multiple range test using multi-way ANOVA (*n* = 18). The disease incidences (%) were determined at 5 days after inoculation with the six isolates. Control represents the fruits inoculation with sterile water but not with the isolates.

**Table 2:** Antagonistic activity of *Bacillus subtilis* JK-14 against *Alternaria tenuis* and *Botrytis cinerea*.

Treatments	Inhibitory Rates (%)	
	*Alternaria tenuis*	*Botrytis cinerea*
*Bacillus subtilis* JK-14	81.32 ± 2.11 b	83.45 ± 1.54 a
Control	-	-

Data are means ± standard error of replicates and those in a column followed by different letters are significantly different at *p* < 0.05, based on Duncan’s new multiple range test using multi-way ANOVA (*n* = 12). The inhibitory rates (%) were determined at 5 days after inoculation with pathogens of *Alternaria tenuis* and *Botrytis cinerea*. Control represents the media inoculation with *Alternaria tenuis* or *Botrytis cinerea* but not with *Bacillus subtilis* JK-14.

**Table 3:** Effect of *Bacillus subtilis* JK-14 on disease incidences and lesion diameters of fresh peach fruits after inoculation with *Alternaria tenuis* and *Botrytis cinerea*.

Treatments	*Alternaria tenuis*		*Botrytis cinerea*	
	Disease Incidences (%)	Lesion Diameters (mm)	Disease Incidences (%)	Lesion Diameters (mm)
*Bacillus subtilis* JK-14	14.8 ± 3.40 b	3.0 ± 0.06 d	14.1 ± 3.40 b	3.2 ± 0.16 d
Control	94.8 ± 1.29 a	12.0 ± 0.12 c	92.6 ± 2.57 a	13.1 ± 0.17 bc

Data are means ± standard error of replicates and those in a column followed by different letters are significantly different at *p* < 0.05, based on Duncan’s new multiple range test using multi-way ANOVA (*n* = 18). The disease incidences (%) and lesion diameters (mm) were determined at 5 days after inoculation with the pathogens. Control represents the peach fruits inoculation with *Alternaria tenuis* or *Botrytis cinerea* but not with *Bacillus subtilis* JK-14.

**Table 4:** Effect of different formulations of *Bacillus subtilis* JK-14 on disease incidences and lesion diameters of mature peach fruits after inoculation with *Alternaria tenuis.*

Concentrations (CFU mL^−1^)	Disease Incidences (%)		Lesion Diameters (mm)	
	FLBC	BCS	FLBC	BCS
1 × 10^9^	47.41 ± 1.28 c	45.18 ± 3.40 cd	6.13 ± 0.13 c	5.69 ± 0.08 d
1 × 10^8^	38.52 ± 3.39 d	36.30 ± 3.40 de	5.61 ± 0.06 d	5.16 ± 0.16 e
1 × 10^7^	18.52 ± 2.56 f	14.82 ± 3.40 f	3.79 ± 0.08 fg	3.06 ± 0.06 g
1 × 10^6^	34.07 ± 3.40 e	31.11 ± 2.22 e	4.95 ± 0.06 e	4.11 ± 0.09 f
1 × 10^5^	65.92 ± 2.60 b	61.48 ± 3.39 b	7.36 ± 0.14 b	6.72 ± 0.19 bc
Control	92.59 ± 1.28 a		11.95 ± 0.09 a	

Data are means ± standard error of replicates and those in a column followed by different letters are significantly different at *p* < 0.05, based on Duncan’s new multiple range test using multi-way ANOVA (*n* = 18). The disease incidences (%) and lesion diameters (mm) were determined at 5 days after inoculation with the pathogen. Control represents the peach fruits inoculation with *Alternaria tenuis* but not with *Bacillus subtilis* JK-14. FLBC represents the fermentation liquid with bacterial cells; BCS represents the bacterial cells suspension. CFU represents colony forming unit.

**Table 5:** Effect of different formulations of *Bacillus subtilis* JK-14 on disease incidences and lesion diameters of mature peach fruits after inoculation with *Botrytis cinerea.*

Concentrations (CFU mL^−1^)	Disease Incidences (%)		Lesion Diameters (mm)	
	FLBC	BCS	FLBC	BCS
1 × 10^9^	44.44 ± 2.23 c	43.70 ± 3.40 c	6.03 ± 0.19 c	5.74 ± 0.22 c
1 × 10^8^	39.26 ± 1.28 d	36.30 ± 1.28 e	5.37 ± 0.25 d	5.13 ± 0.15 d
1 × 10^7^	17.78 ± 2.22 h	14.07 ± 3.40 i	3.74 ± 0.16 f	3.19 ± 0.16 g
1 × 10^6^	28.15 ± 1.28 f	24.44 ± 2.23 g	4.80 ± 0.17 e	4.22 ± 0.16 e
1 × 10^5^	62.22 ± 2.22 b	60.00 ± 2.22 b	7.15 ± 0.08 b	6.66 ± 0.34 b
Control	92.59 ± 2.57 a		13.11 ± 0.17 a	

Data are means ± standard error of replicates and those in a column followed by different letters are significantly different at *p* < 0.05, based on Duncan’s new multiple range test using multi-way ANOVA (*n* = 18). The disease incidences (%) and lesion diameters (mm) were determined at 5 days after inoculation with the plant pathogen. Control represents the peach fruits inoculation with *Botrytis cinerea* but not with *Bacillus subtilis* JK-14. FLBC represents the fermentation liquid with bacterial cells; BCS represents the bacterial cells suspension. CFU represents colony forming unit.
